# Association between childhood infection, serum inflammatory markers and intelligence: findings from a population-based prospective birth cohort study

**DOI:** 10.1017/S0950268817002710

**Published:** 2017-12-04

**Authors:** N. MACKINNON, S. ZAMMIT, G. LEWIS, P. B. JONES, G. M. KHANDAKER

**Affiliations:** 1 Department of Psychiatry, University of Cambridge, Cambridge, UK; 2 Department of Public Health and Primary Care, University of Cambridge, Cambridge, UK; 3 Centre for Mental Health, Addiction and Suicide Research, School of Social and Community Medicine, University of Bristol, Bristol, UK; 4 Division of Psychological Medicine and Clinical Neurosciences, MRC Centre for Neuropsychiatric Genetics and Genomics, Cardiff University, Cardiff, UK; 5 Division of Psychiatry, University College London, London, UK; 6 Cambridgeshire and Peterborough NHS Foundation Trust, Cambridge, UK

**Keywords:** Birth cohort study, C-reactive protein, infection, inflammatory markers, interleukin 6, intelligence quotient

## Abstract

A link between infection, inflammation, neurodevelopment and adult illnesses has been proposed. The objective of this study was to examine the association between infection burden during childhood – a critical period of development for the immune and nervous systems – and subsequent systemic inflammatory markers and general intelligence. In the Avon Longitudinal Study of Parents and Children, a prospective birth cohort in England, we examined the association of exposure to infections during childhood, assessed at seven follow-ups between age 1·5 and 7·5 years, with subsequent: (1) serum interleukin 6 and C-reactive protein (CRP) levels at age 9; (2) intelligence quotient (IQ) at age 8. We also examined the relationship between inflammatory markers and IQ. Very high infection burden (90+ percentile) was associated with higher CRP levels, but this relationship was explained by body mass index (adjusted odds ratio (OR) 1·19; 95% confidence interval (CI) 0·95–1·50), maternal occupation (adjusted OR 1·23; 95% CI 0·98–1·55) and atopic disorders (adjusted OR 1·24; 95% CI 0·98–1·55). Higher CRP levels were associated with lower IQ; adjusted *β* = −0·79 (95% CI −1·31 to −0·27); *P* = 0·003. There was no strong evidence for an association between infection and IQ. The findings indicate that childhood infections do not have an independent, lasting effect on circulating inflammatory marker levels subsequently in childhood; however, elevated inflammatory markers may be harmful for intellectual development/function.

## INTRODUCTION

Infection is both a common and burdensome disease globally. The World Health Organization (WHO) estimated that infectious diseases contributed 301 million disability-adjusted life years in 1 year alone, corresponding to 19·8% of the total burden [1]. The burden of infection is primarily a function of premature death, as mild/moderate infections are thought to have few long-term consequences [2]. However, recent evidence suggests that there are latent consequences of childhood infection that significantly increase its overall burden [3, 4]. Long-term effects of infection may include subtle changes to the immune and nervous systems that, in turn, have adverse health consequences. Upon exposure to an infectious pathogen, the body initiates a systemic inflammatory response due to activation of the innate immune system. Serum concentrations of inflammatory markers, such as interleukin-6 (IL-6) and C-reactive protein (CRP), increase transiently then return to baseline after the infection subsides [5]. However, some individuals exhibit chronic low-grade elevation in circulating IL-6 and CRP levels, which has previously been associated with various illnesses. Prospective cohort studies indicate a correlation between elevated levels of IL-6/CRP and subsequent risk of physical and neuropsychiatric illnesses such as cancers, coronary heart disease, type 2 diabetes, Alzheimer's disease, depression and psychosis [6–12].

The cause of chronically elevated IL-6 and CRP is not well understood. Compared to the initiation of serum spikes during an acute infection, relatively little is known about the molecular factors that return the levels to baseline or maintain chronic, low-grade elevation [13]. Repeated infections during early childhood – a critical period of development for the immune system – would cause multiple spikes in serum IL-6 and CRP, which might programme the immune system for persistent low-grade immune activation. This idea is consistent with the developmental programming hypothesis by David Barker [14] that posits that exposure to certain risk factors during critical periods of development may increase risks of chronic diseases of adult life by programming/altering specific physiological systems. There is evidence that repeated exposure to *Helicobacter pylori* can cause chronic gastritis, a local inflammation [13]. However, no studies to date have examined the effects of repeated exposure to common infections on circulating IL-6 and CRP levels in children, which are typical markers of chronic, low-grade systemic inflammation.

Repeated infection and elevated inflammatory markers are implicated in cognition and neurological disorders. Prenatal infection is associated with neurodevelopmental brain disorders such as schizophrenia, bipolar disorder and autism [15, 16]. In children, exposure to infection and elevated inflammatory markers are associated with risk of impaired neurodevelopment [17]. Exposure to meningitis and other central nervous system (CNS) infections are associated with impaired general intelligence and poor school performance [3, 4]. In older adults, exposure to infections are associated with accelerated cognitive decline and dementia [18], which may be mediated by elevated inflammatory markers [19]. However, most previous studies have looked at specific infections, rather than overall infection burden in childhood, and few studies have concerned neurodevelopment as measured by general intelligence in childhood [20]. Therefore, studies testing the association between exposure to common infections in childhood, IQ and levels of inflammatory markers are required.

We have carried out a longitudinal study to examine the association between exposures to infections during childhood between 1·5 and 7·5 years and subsequent serum concentrations of inflammatory markers (i.e. IL-6 and CRP) at age 9 years and general intelligence (i.e. IQ) at age 8 years. We also tested the association between serum inflammatory markers and IQ. We have examined the effect of confounding factors on these associations, as for example, both infection and IQ are correlated with socio-economic status. In addition, we have carried out secondary analyses to explore the effect of sex and atopic disorders on these associations. The analyses presented here are based on repeated measures of childhood infection in the Avon Longitudinal Study of Parents and Children (ALSPAC), a prospective general population-based birth cohort from England.

## METHODS

### Description of cohort and sample

**Fig. 1.**
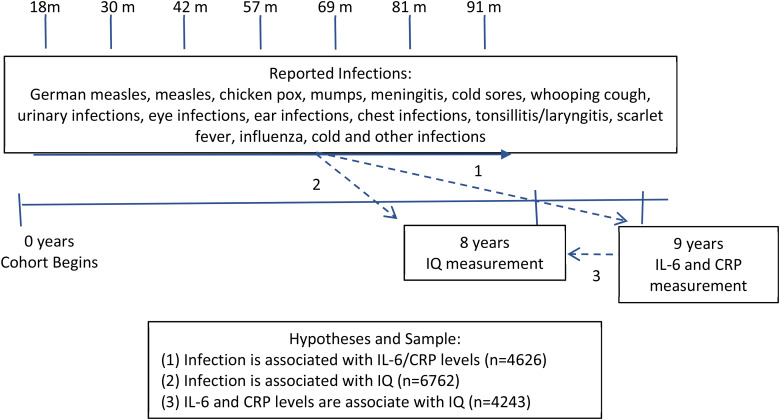
Timeline of data collection, associations tested and sample size.

The ALSPAC birth cohort comprises 14 062 live births from pregnant women resident in county Avon, a geographically defined region in southwest of England, with expected dates of delivery between April 1991 and December 1992 [21]. The study website contains details of all available data through a fully searchable ‘data dictionary’ (http://www.bris.ac.uk/alspac/researchers/data-access/data-dictionary/). Parents completed regular postal questionnaires about aspects of their child's health and development from birth. Since age 7, the children attended an annual assessment clinic during which they participated in various face-to-face interviews, physical and clinical tests [22]. Our study is based on infection data collected through postal questionnaires until age 7·5 years. Figure 1 shows the timeline of data collection and sample size for specific analyses, which included up to 6762 participants.

Ethical approval for the study was obtained from ALSPAC Ethics and Law Committee and the Local Research Ethics Committees.

### Assessment of childhood infection between age 1·5 and 7·5 years

Caregivers filled out seven questionnaires about common childhood infection at ages 18, 30, 42, 57, 69, 81 and 91 months old. In total, 11 786 children had infection data for at least one time point. Caregivers were asked to check ‘yes’ or ‘no’ if their child had any of the infections listed: German measles, measles, chicken pox, mumps, meningitis, cold sores, whooping cough, urinary infections, eye infections, ear infections, chest infections, tonsillitis/laryngitis, scarlet fever, influenza, cold and ‘other’. If a child had missing data on infection at all time points, they were excluded. If infection data were missing for at least one but not all time points, the missing values were replaced with zero assuming no infection at that age in order to maximise sample size. Infection was used as a continuous variable, and then to further explore each association, infection count was summed and then divided categorically as low (below median), medium (50–75th percentile), high (76–90th percentile) and very high burden (90th percentile and above). The categories correspond to 0–4, 5–6, 7–9 and 10+ infections, respectively. These thresholds were chosen to appropriately capture the positive skew of infection distribution. The distribution of infection frequency is presented in online Supplementary Fig. S1.

### Assessment of IQ at age 8 years

IQ was measured by the Wechsler Intelligence Scale for Children (WISC) III, Third UK edition [23]. In total, 7153 children had complete IQ data. A shortened version of the test was applied by trained psychologists, whereby alternate items (always starting with item number 1 in the standard form) were used for all 10 subtests except for the coding subtest which was administered in its standard form. Use of the shortened version reduced the length of assessment, so the children were less likely to tire. This approach has been successfully used in other studies [24, 25]. IQ data obtained using this method have shown robust correlations with neurodevelopmental disorders, and other concurrent neurocognitive measures such as working memory, short-term memory and socio-demographic factors such as social class [24, 26]. Notably, IQ was measured prior to serum inflammatory markers but given the WISC-III shows robust test–retest reliability during childhood (6–13 years), we have used it as an outcome in this analysis [27].

### Assessment of inflammatory markers at age 9 years

Serum IL-6 and CRP levels were assessed in non-fasting blood samples collected at 9 years of age. Samples were stored at −80 °C for a median 7·5 years; there was no evidence of freeze–thaw during storage. IL-6 was measured by ELISA (R&D systems, Abingdon, UK) and CRP was measured using automated particle-enhanced immunoturbidimetric assay (Roche, UK, Welwyn Garden City, UK). The minimum detection limit was 0·007 pg/ml and 0·01 mg/l for IL-6 and CRP, respectively. IL-6 and CRP levels were log-transformed to adjust for a positive skew. In total, 4942 children had complete inflammatory marker data after excluding those with self-reported infections around blood collection.

### Assessment of covariates

We included sex, body mass index (BMI), maternal occupation, atopic disorders and household crowding as potential confounders. BMI (weight in kg divided by height in metres squared) was assessed at the time of blood collection for inflammatory marker assay at age 9 years, which was treated as a continuous variable. Maternal occupation was reported by the caregiver during pregnancy (~23–32 weeks gestation) per the 1991 UK Office of Population Censuses and Surveys classification (professional, managerial, skilled non-manual, skilled manual, semi-skilled manual and unskilled manual occupations), which was coded into a binary variable (manual and non-manual occupations). Atopic disorders were obtained by parent report of physician diagnosis of eczema, asthma or both at age 9. For stratified analyses, atopic disorders were coded into a binary variable (any disorder, no disorder). Household crowding is a ratio of the number of people living in the house per room (excluding kitchen), which was calculated by self-reported data at 23–32 weeks gestation.

### Statistical analysis

Baseline characteristics were compared across categories of infection burden using *χ*^2^ test for categorical variable (sex, maternal occupation, atopic disorders) and one-way analysis of variance (ANOVA) for continuous variable (BMI and household crowding).

Three separate analyses were conducted to investigate the associations between infection, IQ and inflammatory markers (Fig. 1). First, we carried out logistic regression to examine the odds ratio (OR) for being in the top third of IL-6 distribution (>1·14 pg/ml) at age 9 years for children with the highest infection burden compared with low infection burden (*N* = 4626). The same was done for CRP (the threshold for the top third of the CRP distribution was 1·39 mg/l) (*N* = 4636). In addition, the association between infection and inflammatory markers was assessed by linear regression using natural log-transformed values of IL-6 and CRP (*N* = 4626 and 4636, respectively).

Second, linear regression was used to test the association between infection and IQ (*N* = 6762). To further explore the relationship, mean differences in total IQ scores across quantiles of infection are presented and Wald's test was used for significance (*N* = 6762). For analyses with infection data, we included all participants with data at least one time point and assumed that all missing data points were equivalent to zero infection. To test this assumption, we further conducted sensitivity analysis restricting the sample to participants with complete information on infection from all seven time points between age 1·5 and 7·5 years (*N* = 3222–4691 depending on the analysis).

Third, natural log-transformed IL-6 and CRP levels were regressed on IQ level to ascertain whether elevated inflammatory markers predict IQ (*N* = 4243 and *N* = 4253, respectively).

All analyses were subsequently adjusted for sex, BMI, household crowding, atopic status and maternal occupation. In all cases, when associations were not significant upon adjustment, the analysis was repeated with each confounder individually to ascertain which variables account for the non-significant result.

Finally, to further explore each relationship, each analysis was repeated stratified by sex and atopic status (binary variable). Secondary analysis was also conducted in which infection burden was categorised as quartiles rather than by the percentile thresholds previously mentioned.

## RESULTS

### Baseline characteristics

The sample was predominantly white, and more affluent than the national average as demonstrated by the prevalence of non-manual occupations (Table 1). There were significant differences in BMI, sex, maternal occupation, atopic diagnosis and household crowding across categories of infection burden. These variables were included as confounders in the subsequent analyses. The number of infections between ages 1·5 and 7·5 years ranged from 0 to 22; the median was 4 (IQR = 2–6). CRP levels at age 9 years ranged from 0·01 to 67·44 mg/l; the median CRP level was 0·21 (IQR = 0·11–0·54). IL-6 levels ranged from 0·01 to 20·10 pg/ml; the median IL-6 level was 0·81 (IQR = 0·50–1·42). IQ scores at age 8 years ranged from 45 to 151; the median was a score of 104 (IQR = 93–116) (see online Supplementary Fig. S2).

**Table 1.** Baseline characteristics of the study sample

Group/characteristic	Low	Medium	High	Very high	*P*-value*
Number of infections (percentile of infection distribution)	0–4 (0–49)	5–6 (50–75)	7–9 (76–90)	10–22 (⩾90)	
Total participants, No. (%)	6371 (54·1)	2480 (21·0)	1988 (16·9)	947 (8·0)	
BMI, mean (sd)	17·2 (2·9)	17·1 (2·8)	17·3 (2·8)	17·3 (2·8)	0·019
Sex (male), No. (%)	3359 (52·7)	1262 (50·9)	991 (49·8)	464 (49·0)	0·033
Maternal occupation (manual), No. (%)	1018 (21·8)	349 (17·0)	241 (14·5)	144 (17·9)	<0·001
Atopy, No (%)					<0·001
Has asthma	362 (12·0)	252 (13·5)	288 (18·0)	144 (18·3)	
Has eczema	361 (12·0)	246 (13·2)	205 (12·8)	109 (13·8)	
Has both	127 (4·2)	117 (6·3)	155 (9·7)	125 (15·9)	
Has neither	2170 (71·8)	1247 (67·0)	950 (59·4)	409 (52·0)	
Household crowding, mean (sd)	2·2 (1·1)	2·0 (1·0)	1·9 (1·0)	2·0 (1·0)	<0·001

*P-values correspond to the Pearson's *χ*^2^ test for sex, atopic disorder and maternal occupation, and for an ANOVA test for BMI and house crowding.

### Association between infection and systemic inflammatory markers

After adjusting for potential confounders, there was no evidence that children with the highest infection burden, compared with children with low infection burden, were more likely to have high IL-6 (adjusted OR 1·13; 95% confidence interval (CI) 0·88–1·47) or high CRP (adjusted OR 1·02; 95% CI 0·77–1·34) levels at age 9 years (Table 2). Addition of each confounder separately to the unadjusted model suggested that BMI, maternal occupation and atopic status most confound the association between very high infection burden and CRP. The results were no longer significant after adjusting for BMI (adjusted OR 1·19; 95% CI 0·95–1·50); maternal occupation (adjusted OR 1·23; 95% CI 0·98–1·55); atopy (adjusted OR 1·24; 95% CI 0·98–1·55).

**Table 2.** Odds ratio (OR) for high serum IL-6 and CRP levels at age 9 years for infection burden between age 1·5 and 7·5 years

Inflammatory marker	Infection burden between 1·5 and 7·5 years (no. of infections)	Sample size, no.	In top third of inflammatory marker distributions No. (%)	OR (95% CI) for being in the top third of inflammatory marker distribution at 9 years	
IL-6				Unadjusted	Adjusted*
	Low (0–4)	1970	626 (31·78)	1·00 (reference)	1·00 (reference)
	Medium (5–6)	1204	402 (33·39)	1·08 (0·92–1·25)	1·07 (0·88–1·29)
	High (7–9)	963	315 (32·71)	1·04 (0·88–1·23)	0·98 (0·80–1·20)
	Very high (10–22)	489	183 (37·42)	1·28 (1·04–1·58)	1·13 (0·88–1·47)
CRP					
	Low (0–4)	1973	615 (31·17)	1·00 (reference)	1·00 (reference)
	Medium (5–6)	1206	417 (34·58)	1·17 (1·00–1·36)	1·21 (0·99–1·49)
	High (7–9)	967	322 (33·30)	1·10 (0·94–1·30)	1·06 (0·85–1·31)
	Very high (10–22)	490	179 (36·53)	1·27 (1·03–1·56)	1·02 (0·77–1·34)

* Adjusted for sex, BMI, maternal occupation, atopic disorder and household crowding.

The analysis was repeated using infection as a continuous variable. Linear regression found no evidence for an association between childhood infection burden and subsequent serum IL-6 levels or CRP levels at age 9 years (see online Supplementary Table S1a). Furthermore, sensitivity analyses based on participants with complete data on infection exposure between age 1·5 and 7·5 years showed similar results when infection was coded as both a continuous and categorical variable (see online Supplementary Tables S1b and S2).

### Association between infection and IQ

The mean IQ in high infection burden group was higher compared with low infection burden group (mean difference = 1·70 (95% CI 0·45–2·95)), but there was no observable dose response (see online Supplementary Table S3). Moreover, analysis based on only complete cases did not confirm this relationship (mean difference = −0·03 (95% CI −1·92 to 1·86)).

The analysis was repeated using infection as a continuous variable. Linear regression showed that there was a significant increase in IQ with increasing infection burden in the adjusted model (*β* = 0·22 (95% CI 0·07–0·37)) (Table 3). However, sensitivity analyses based on complete cases found no association between infection burden and IQ (*P* = 0·694) (see online Supplementary Table S4).

**Table 3.** Association between infection burden between 1·5 and 7·5 years and total IQ score at age 8 years

Unadjusted analysis			Adjusted analysis*		
Sample	ΔIQ per infection (95% CI)	*P*-value	Sample	ΔIQ per infection (95% CI)	*P*-value
6762	0·41 (0·28–0·54)	<0·001	4392	0·22 (0·07–0·37)	0·008

* Adjusted for sex, BMI, maternal occupation, atopic disorder and household crowding.

### Association between systemic inflammatory markers and IQ

Increasing CRP levels, but not IL-6, were associated with lower IQ in the adjusted model (*β* = −0·79 (−1·31 to −0·27). This corresponds to about 0·8% decrease in IQ per one point increase in CRP (Table 4). The association for IL-6 was attributed to socio-economic factors (house crowding, maternal occupation) as the significance was lost after adjusting for these variables. The *β* was −0·48 (95% CI −1·06 to 0·89) after adjusting for household crowding, and *β* was −0·43 (95% CI −1·04 to 0·18) after adjusting for maternal occupation.

**Table 4.** Association between serum IL-6 and CRP levels and total IQ score

Inflammatory marker	Unadjusted analysis			Adjusted analysis*		
	*N*	ΔIQ per 1 pg/ml (IL-6) or mg/L (CRP) (95% CI)	*P*-value	*N*	ΔIQ per 1 pg/ml (IL-6) or mg/L (CRP) (95% CI)	*P*-value
IL-6	4243	−0·80 (−1·37 to −0·24)	0·005	2960	−0·26 (−0·93 to 0·41)	0·446
CRP	4253	−0·89 (−1·30 to −0·49)	<0·001	2967	−0·79 (−1·31 to −0·27)	0·003

* Adjusted for sex, BMI, maternal occupation, atopic disorder and household crowding.

### Results for secondary analyses

To explore any subgroup effects, we re-analysed the main associations, first after stratifying the sample by sex, and second after stratifying the sample by the presence or absence of atopic disorders (asthma and/or eczema). In sex-stratified analyses, the overall pattern of association between (1) infection burden and IL-6/CRP, (2) infection burden and IQ, and (3) IL-6/CRP and IQ in male and female participants were similar to that in the original analysis (see online Supplementary Tables S5–S7).

The overall pattern of association between infection burden and IL-6/CRP was similar between groups with and without atopic disorders. For the association between infection burden and IQ, the effect size attenuated but were similar between groups with and without atopic disorders. Interestingly, the association between CRP and IQ was found to be non-significant after adjusting for confounders only in the atopic group, suggesting the association is only present when asthma and/or eczema are not present (see online Supplementary Tables S9 and S10).

Finally, we repeated the analyses testing between infection burden and IL-6/CRP after re-categorising the infection burden variable into quartiles (see online Supplementary Table S11). Using this variable, no significant association between infection burden and inflammatory markers were observed.

## DISCUSSION

We have examined prospective associations between exposure to common and serious infections during childhood and subsequent serum levels of IL-6, CRP and general intelligence. First, higher infection burden was associated with higher levels of IL-6 and CRP, but this association was explained by body mass, atopic disorder and socio-economic status. The results indicate that socio-economic and physical factors, rather than repeated infections in past, have an important effect on inflammatory marker concentrations in childhood. Second, we report that elevated IL-6 and CRP were significantly associated with lower IQ scores, suggesting there may be a detrimental effect of high inflammatory markers on general intelligence. Evidence for an association between IQ and CRP persisted after controlling for potential confounders. Previous studies have reported an association between infection, elevated inflammatory marker levels and cognitive function in older adults [18, 28]. A proteomics study of Nepalese children has reported that general intelligence is correlated with six inflammatory proteins [29], but to our knowledge, this is the first study to report an association between CRP and general intelligence in childhood. There was no strong evidence for an association between infection burden and IQ. This is different from a Danish cohort study that reported an association between severe infections requiring hospitalisation and lower IQ [30]. The current study is based on mostly common childhood infections, which might explain this difference. Our finding could be also due to reporting bias, for example, more educated mothers may be more likely to report infections or less likely to omit infection data (see below).

Our study contributes epidemiological evidence to support growing molecular evidence demonstrating links between cytokines and central nervous system (CNS) [31]. Peripheral inflammation can cause CNS inflammation leading to increased neurotransmitter metabolism and impaired neuroplasticity [15, 19, 32]. The subsequent effects are observed clinically, where immune dysregulation correlates with behavioural changes. Elevated IL-6 and/or CRP has been associated with depression, autism, schizophrenia and related psychoses [31, 33]. Our study extends the literature by showing that low-grade systemic inflammation is associated with general intelligence in early childhood, lending support to the idea that inflammation may contribute to impaired neurodevelopment, which is central to the origins of many of these psychiatric illnesses.

Strengths of the study includes a large, population-based sample, longitudinal design, repeated data of childhood infection collected prospectively, and robust measures of both IQ and serum inflammatory markers subsequently in childhood. However, the results should be interpreted in the context of several limitations. First, infection was assumed to be zero if data were missing. We used sensitivity analysis to verify our results, but given uneven attrition of certain demographics, selection bias is possible. Second, we could not account for ethnicity in our analysis. Past evidence suggests that there is wide variations in the correlation between ethnicity IL-6 and CRP levels, and given the non-white participants made up only 2% of the total sample, we felt its inclusion would not provide any meaningful comparison across categories of infection [34, 35]. Third, socio-economic factors were measured by maternal occupation and household crowding. We acknowledge that this might miscategorise subjects with, for example, a working father and non-working mother. Fourth, infection was measured by parental report rather than medical records, making it vulnerable to varying reporting practices [36]. For example, more educated parents may report more infections, falsely strengthening the association between infection and high IQ. Similarly, we did not have information on infection severity, making this variable vulnerable to reporting practices, as some caregivers may have omitted minor infections. Lastly, IQ was measured prior to inflammatory markers despite being an outcome in our analysis. WISC-III has specifically shown good long-term test–retest reliability during childhood (mean age 9·15, range 6–15) when retested a mean of 2·83 years later (range 0·5–6·2 years) [27]. Nevertheless, we acknowledge it is also possible that IQ may influence risk of infection.

As such, our findings identify important research priorities. We show that socio-economic disadvantage, body mass and atopy explain the relationship between infection and elevated systemic inflammatory marker levels previously reported to be associated with neurological health consequences. Identifying how social disadvantage leads to elevated inflammatory markers is integral to mitigating these consequences. In the future, studies should examine the effect of specific infections. For instance, viral infections are protective for immune disorders such as asthma, allergy and atopy [37, 38], and therefore it is plausible that the relationship between infection and inflammatory markers may vary by specific types of infection. It is possible that individuals with disadvantaged socio-economic backgrounds are exposed to more detrimental infections. Understanding the key mediators of the link between socio-economic status and inflammatory makers is critical to taking preventative measures to mitigate adverse consequences of infection. Such work would help to minimise the cost of socio-economic disadvantage on health outcomes.

## CONCLUSION

The relationship between infection in childhood and subsequent concentrations of inflammatory markers is explained by socio-economic and physical factors. However, increased inflammatory marker levels, particularly CRP, is associated with reduced general intelligence, which persist after controlling for socio-economic and other confounders. These findings suggest that low-grade systemic inflammation might be detrimental for brain development and function. Further research is needed to identify mediators of the relationship between infection and inflammatory markers.

## Supplementary material

For supplementary material accompanying this paper visit https://doi.org/10.1017/S0950268817002710. click here to view supplementary material
