# Supplementary material for: Association between childhood infection, serum inflammatory markers and intelligence: findings from a population-based prospective birth cohort study
Source: Epidemiol Infect. 2017 Dec 4;146(2):256–64. doi: 10.1017/S0950268817002710 (PMC5851035; doi:10.1017/S0950268817002710)
Supplement: Supplementary file 1 [file S0950268817002710sup001.docx]

**Epidemiology and Infection**

Association between Childhood Infection, Serum Inflammatory Markers and Intelligence: findings from a population-based prospective birth cohort study

N. MACKINNON, S. ZAMMIT, G. LEWIS, P.B. JONES, G.M. KHANDAKER

**Tables**

Online Supplementary Table S1:

1. Linear association between infection burden and serum IL6 and CRP levels.

| Inflammatory Marker | Unadjusted analysis | | | Adjusted analysis1 | | |
| --- | --- | --- | --- | --- | --- | --- |
|  | N | ΔIL-6 or ΔCRP per infection (95% CI) | P-Value | N | ΔIL-6 or ΔCRP per infection (95% CI) | P-Value |
| IL-6 |  |  |  |  |  |  |
| Infections | 4626 | 0.006 (-0.002, 0.0144) | 0.131 | 3251 | 0.006 (-0.003, 0.016) | 0.228 |
| CRP |  |  |  |  |  |  |
| Infections | 4636 | 0.012 (0.001, 0.236) | 0.035 | 3258 | 0.005 (-0.007, 0.018) | 0.443 |

^1^ Adjusted for sex, BMI, maternal occupation, atopic disorders and household crowding

1. Linear association between infection burden and serum IL6 and CRP levels. Only complete cases were used.

| Inflammatory Marker | Unadjusted analysis | | | Adjusted analysis^1^ | | |
| --- | --- | --- | --- | --- | --- | --- |
|  | N | ΔIL-6 or ΔCRP per infection (95%CI) | P-Value | N | ΔIL-6 or ΔCRP per infection (95%CI) | P-Value |
| IL-6 |  |  |  |  |  |  |
| Infections | 3222 | 0.013 (0.003, 0.022) | 0.011 | 2573 | 0.008 (-0.003, 0.20) | 0.150 |
| CRP |  |  |  |  |  |  |
| Infections | 3231 | 0.019 (0.005, 0.033) | 0.006 | 2580 | 0.008 (-0.006, 0.022) | 0.278 |

^1^Adjusted for sex, BMI, maternal occupation, atopic disorders and house crowding

Online Supplementary Table S2: Odds of high serum IL-6 and CRP levels (being in top third of the distribution) as a function of infection burden, presented as low (below median), medium (50-75th percentile), high (76-90th percentile), and very high burden (90th percentile and above). Only complete cases were used.

| Inflammatory Marker | Infection Burden | Sample, No. | In the top third of IL-6 distribution1, No. (%) | OR (95% CI) for being in the top third of IL-6 distribution at 9 years | |
| --- | --- | --- | --- | --- | --- |
|  |  |  |  | Unadjusted | Adjusted^1^ |
| IL-6 |  |  |  |  |  |
|  | Low | 1994 | 631 (31.64) | [reference] | [reference] |
|  | Medium | 626 | 188 (30.03) | 0.93 (0.76, 1.13) | 0.87 (0.70, 1.09) |
|  | High | 325 | 112 (34.46) | 1.14 (0.89, 1.45) | 0.96 (0.72, 1.29) |
|  | Very High | 277 | 108 (38.99) | 1.38 (1.07, 1.79) | 1.26 (0.93, 1.72) |
| CRP |  |  |  |  |  |
|  | Low | 1999 | 615 (30.77) | [reference] | [reference] |
|  | Medium | 629 | 193 (30.68) | 1.00 (0.82, 1.21) | 0.93 (0.73, 1.17) |
|  | High | 329 | 109 (33.44) | 1.13 (.88, 1.45) | 0.90 (0.66, 1.24) |
|  | Very High | 277 | 99 (35.74) | 1.25 (0.96, 1.63) | 1.11 (0.80, 1.54) |

^1^Adjusted for sex, BMI, maternal occupation, atopic disorder and house crowding

Online Supplementary Table S3: Mean IQ score according to category of infection

| Infection Burden  (# of infections) | N | IQ mean (SD) | Mean Differences (95% CI) | Adjusted^1^  Mean Differences (95% CI) |
| --- | --- | --- | --- | --- |
| Low (0-4) | 2853 | 102.79 (16.15) | [reference] | [reference] |
| Medium (5-6) | 1740 | 105.22 (16.47) | 2.43 (1.46, 3.40) | 0.93 (-0.25, 2.11) |
| High (7-9) | 1450 | 106.04 (16.74) | 3.25 (2.12, 4.28) | 1.70 (0.45, 2.95)) |
| Very High (10-22) | 719 | 105.02 (16.67) | 2.23 (0.90, 3.56) | 1.48 (-0.10, 3.06) |
| P-value^2^ |  |  | <0.001 | 0.048 |

^1^Adjusted for sex, BMI, maternal occupation, atopic disorder and house crowding

Online Supplementary Table S4:

(A) Mean IQ score according to category of infection. Only with complete infection data cases were included.

| Infection Category  (# of infections) | N | IQ mean (SD) | Crude  Mean Difference (95% CI) | Adjusted^1^  Mean Difference (95% CI) |
| --- | --- | --- | --- | --- |
| Low (0-4) | 2883 | 105.87 (15.83) | Ref. | Ref. |
| Medium (5-6) | 892 | 106.95 (16.47) | 1.08(-0.13, 2.29) | 0.75 (-0.62, 2.11) |
| High (7-9) | 503 | 106.15 (16.17) | 0.27(-1.25, 1.80) | 0.57 (-1.14, 2.28) |
| Very High (10-22) | 413 | 105.18 (17.24) | -0.69(-2.35,0.97) | -0.03(-1.92, 1.86) |
| P-value^2^ |  |  | 0.225 | 0.694 |

^1^Adjusted for sex, BMI, maternal occupation, atopic disorder and house crowding

^2P^-values are calculated from a Wald’s test

(B) Linear regression between infection and IQ. Only complete infection data cases were included.

| Unadjusted analysis | | | Adjusted analysis^1^ | | |
| --- | --- | --- | --- | --- | --- |
| N | ΔIQ per infection  (95%CI) | P-Value | N | ΔIQ per infection  (95%CI) | P-Value |
| 4691 | 0.56 (-0.97, 0.210) | 0.473 | 3534 | 0.79 (-0.10, 0.26) | 0.382 |

^1^Adjusted for sex, BMI, maternal occupation, atopic disorder and house crowding

Online Supplementary Table S5: Odds ratio (OR) for high serum IL-6 and CRP levels at age 9 years for infection burden between age 1.5 and 7.5 years for male and female participants separately

| Inflammatory Marker/ Group | | Infection Burden between 1.5 and 7.5 years (No. of Infections) | Sample Size, No. | In top third of inflammatory marker distributions  No. (%) | OR (95% CI) for being in the top third of inflammatory marker distribution at 9 years | |
| --- | --- | --- | --- | --- | --- | --- |
|  |  |  |  |  | Unadjusted | Adjusted^1^ |
| IL-6 | | | | | | |
| All Participants | | | | | | |
|  | Low (0-4) | | 1970 | 626 (31.78) | [reference] | [reference] |
|  | Medium (5-6) | | 1204 | 402 (33.39) | 1.08 (0.92, 1.25) | 1.07 (0.88, 1.29) |
|  | High (7-9) | | 963 | 315 (32.71) | 1.04 (0.88, 1.23) | 0.98 (0.80, 1.20) |
|  | Very High (10-22) | | 489 | 183 (37.42) | 1.28 (1.04, 1.58) | 1.13 (0.88, 1.47) |
| Males | | | | | | |
|  | Low (0-4) | | 1039 | 271 (26.08) | [reference] | [reference] |
|  | Medium (5-6) | | 594 | 159 (26.77) | 1.04 (0.82, 1.30) | 1.03 (0.78, 1.37) |
|  | High (7-9) | | 477 | 132 (27.67) | 1.08 (0.85, 1.28) | 1.07 (0.80, 1.44) |
|  | Very High (10-22) | | 240 | 78 (32.50) | 1.36 (1.01, 1.85) | 1.09 (0.74, 1.58) |
| Females | | | | | | |
|  | Low (0-4) | | 931 | 355 (38.13) | [reference] | [reference] |
|  | Medium (5-6) | | 610 | 243 (39.84) | 1.07 (0.87, 1.32) | 1.10 (0.84, 1.43) |
|  | High (7-9) | | 486 | 183 (37.65) | 1.18 (0.89, 1.57) | 0.90 (0.68, 1.20) |
|  | Very High (10-22) | | 249 | 105 (42.17) | 1.18 (0.89, 1.57) | 1.18 (0.83, ,1.68) |
| CRP | | | | | | |
| All Participants | | | | | | |
|  | Low (0-4) | | 1973 | 615 (31.17) | [reference] | [reference] |
|  | Medium (5-6) | | 1206 | 417 (34.58) | 1.17 (1.00, 1.36) | 1.21 (0.99, 1.49) |
|  | High (7-9) | | 967 | 322 (33.30) | 1.10 (9.35, 1.30) | 1.06 (0.85, 1.31) |
|  | Very High (10-22) | | 490 | 179 (36.53) | 1.27 (1.03, 1.56) | 1.02 (0.77, 1.34) |
| Males | | | | | | |
|  | Low (0-4) | | 1041 | 260(24.98) | [reference] | [reference] |
|  | Medium (5-6) | | 596 | 162 (27.18) | 1.12 (0.89, 1.41) | 1.17 (0.87, 1.58) |
|  | High (7-9) | | 480 | 119 (24.79) | 0.99 (0.77, 1.27) | 0.95 (0.69, 1.31) |
|  | Very High (10-22) | | 241 | 64 (26.56) | 1.09 (0.79, 1.49) | 0.86 (0.57, 1.32) |
| Females | | | | | | |
|  | Low (0-4) | | 932 | 355 (38.09) | [reference] | [reference] |
|  | Medium (5-6) | | 610 | 255 (41.80) | 1.17 (0.95, 1.44) | 1.24 0.94, 1.63) |
|  | High (7-9) | | 487 | 203 (41.68) | 1.16 (0.93, 1.45) | 1.13 (0.84, 1.51) |
|  | Very High (10-22) | | 249 | 115 (46.18) | 1.39 (1.05, 1.85) | 1.16 (0.80, 1.68) |

^1^Adjusted for BMI, maternal occupation, atopic disorder and house crowding

Online Supplementary Table S6: Association between infection burden between 1.5 and 7.5 years and total IQ score at age 8 years for male and female participants separately

|  | Unadjusted analysis | | | Adjusted analysis^1^ | | |
| --- | --- | --- | --- | --- | --- | --- |
|  | Sample | ΔIQ per infection (95%CI) | P-Value | Sample | ΔIQ per infection (95%CI) | P-Value |
| All Participants | 6762 | 0.41 (0.28, 0.54) | <0.001 | 4392 | 0.22 (0.07, 0.37) | 0.008 |
| Males | 3371 | 0.47 (0.28, 0.66) | <0.001 | 2177 | 0.23 (-0.01, 0.47) | 0.057 |
| Females | 3391 | 0.36 (0.19, 0.53) | <0.001 | 2215 | 0.20 (-0.01, 0.41) | 0.067 |

^1^Adjusted for BMI, maternal occupation, atopic disorder and house crowding

Online Supplementary Table S7: Association between serum IL-6 and CRP levels and total IQ score for male and female participants separately

|  | Unadjusted analysis | | | Adjusted analysis^1^ | | |
| --- | --- | --- | --- | --- | --- | --- |
| Inflammatory Marker | N | ΔIQ per 1pg/ml (IL-6) or  mg/L (CRP) (95%CI) | P-Value | N | ΔIQ per 1pg/ml (IL-6) or  mg/L (CRP) (95%CI) | P-Value |
| All participants | | | | | | |
| IL-6 | 4243 | -0.80 (-1.37, - 0.24) | 0.005 | 2960 | -0.26(-0.93, 0.41) | 0.446 |
| CRP | 4253 | -0.89 (-1.30, -0.49) | <0.001 | 2967 | -0.79(-1.31, -0.27) | 0.003 |
| Males | | | | | | |
| IL-6 | 2123 | -0.88 (-1.68, 0.07) | 0.033 | 1502 | -0.47 (-1.43, 0.48) | 0.330 |
| CRP | 2131 | -0.74 (-1.34, -0.15) | 0.014 | 1509 | -0.82 (-1.58, -0.061) | 0.034 |
| Females | | | | | | |
| IL-6 | 2120 | -0.71 (-1.51, 0.10) | 0.087 | 1458 | 0.01 (-0.93, 0.95) | 0.984 |
| CRP | 2122 | -1.06 (-1.63, -0.50) | <0.001 | 1458 | -0.75 (-1.47, -0.03) | 0.041 |
|  | | | | | | |

^1^Adjusted for BMI, maternal occupation, atopic disorder and house crowding

Online Supplementary Table S8: Odds ratio (OR) for high serum IL-6 and CRP levels at age 9 years for infection burden between age 1.5 and 7.5 years, stratified by atopic disorder status

| Inflammatory Marker/ Group | Infection Burden between 1.5 and 7.5 years (No. of Infections) | | Sample Size, No. | In top third of inflammatory marker distributions  No. (%) | OR (95% CI) for being in the top third of inflammatory marker distribution at 9 years | |
| --- | --- | --- | --- | --- | --- | --- |
| IL-6 |  | |  |  | Unadjusted | Adjusted^1^ |
| All Participants | | | | | | |
|  | Low (0-4) | 1970 | | 626 (31.78) | [reference] | [reference] |
|  | Medium (5-6) | 1204 | | 402 (33.39) | 1.08 (0.92, 1.25) | 1.07 (0.88, 1.29) |
|  | High (7-9) | 963 | | 315 (32.71) | 1.04 (0.88, 1.23) | 0.98 (0.80, 1.20) |
|  | Very High (10-22) | 489 | | 183 (37.42) | 1.28 (1.04, 1.58) | 1.13 (0.88, 1.47) |
| Has Atopic Disorder | | | | | | |
|  | Low (0-4) | 427 | | 137 (32.08) | [reference] | [reference] |
|  | Medium (5-6) | 353 | | 128 (36.26) | 1.20 (0.89, 1.62) | 1.18 (0.84, 1.67) |
|  | High (7-9) | 327 | | 107 (32.72) | 1.03 (0.76, 1.40) | 1.00 (0.70, 1.43) |
|  | Very High (10-22) | 218 | | 88 (40.37) | 1.43 (1.02, 2.01) | 1.39 (0.95, 2.05) |
| No Atopic Disorder | | | | | | |
|  | Low (0-4) | 1135 | | 351 (30.93) | [reference] | [reference] |
|  | Medium (5-6) | 701 | | 226 (32.24) | 1.06 (0.87, 1.30) | 1.02 (0.81, 1.29) |
|  | High (7-9) | 553 | | 180 (32.55) | 1.08 ().87, 1.34) | 1.00 (0.78, 1.28) |
|  | Very High (10-22) | 226 | | 78 (34.51) | 1.18 (0.87, 1.59) | 0.99 (0.70, 1.41) |
| CRP | | | | | | |
| All Participants | | | | | | |
|  | Low (0-4) | 1973 | | 615 (31.17) | [reference] | [reference] |
|  | Medium (5-6) | 1206 | | 417 (34.58) | 1.17 (1.00, 1.36) | 1.21 (0.99, 1.49) |
|  | High (7-9) | 967 | | 322 (33.30) | 1.10 (9.35, 1.30) | 1.06 (0.85, 1.31) |
|  | Very High (10-22) | 490 | | 179 (36.53) | 1.27 (1.03, 1.56) | 1.02 (0.77, 1.34) |
| Has Atopic Disorder | | | | | | |
|  | Low (0-4) | 429 | | 151 (35.20) | [reference] | [reference] |
|  | Medium (5-6) | 354 | | 134 (37.85) | 1.12 (0.84, 1.50) | 1.34 (0.93, 1.93) |
|  | High (7-9) | 329 | | 112 (34.04) | 0.95 (0.70, 1.29) | 1.03 (0.71, 1.49) |
|  | Very High (10-22) | 218 | | 80 (36.70) | 1.07 (0.76, 1.50) | 0.99 (0.65, 1.51) |
| No Atopic Disorder | | | | | | |
|  | Low (0-4) | 1136 | | 329 (28.96) | [reference] | [reference] |
|  | Medium (5-6) | 702 | | 230 (32.76) | 1.20 (0.98, 1.46) | 1.16 (0.91, 1.48) |
|  | High (7-9) | 554 | | 181 (32.67) | 1.19 (0.96, 1.48) | 1.09 (0.84, 1.41) |
|  | Very High (10-22) | 227 | | 82 (36.12) | 1.39 (1.03, 1.87) | 1.11 (0.76, 1.60) |

^1^Adjusted for sex, BMI, maternal occupation, and household crowding

Online Supplementary Table S9 Association between infection burden between 1.5 and 7.5 years and total IQ score at age 8 years, stratified by atopic disorder status

|  | ----------Unadjusted analysis---------- | | | ----------Adjusted analysis^1^---------- | | |
| --- | --- | --- | --- | --- | --- | --- |
|  | Sample | ΔIQ per infection (95%CI) | P-Value | Sample | ΔIQ per infection (95%CI) | P-Value |
| All Participants | 6762 | 0.41 (0.28, 0.54) | <0.001 | 4392 | 0.22 (0.07, 0.37) | 0.008 |
| Has Atopic Disorder | 1942 | 0.28 (0.06, 0.51) | 0.014 | 1516 | 0.23 (-0.02, 0.48) | 0.074 |
| No Atopic Disorder | 3626 | 0.23 (0.04, 0.41) | 0.017 | 2876 | 0.17 (-0.02, 0.39) | 0.073 |

^1^Adjusted for sex, BMI, maternal occupation, and household crowding

Online Supplementary Table S10: Association between serum IL-6 and CRP levels and total IQ score, stratified by atopic disorder status

|  | ----------Unadjusted analysis---------- | | | ----------Adjusted analysis^1^---------- | | |
| --- | --- | --- | --- | --- | --- | --- |
| Inflammatory Marker/ Group | N | ΔIQ per 1pg/ml (IL-6) or  mg/L (CRP) (95%CI) | P-Value | N | ΔIQ per 1pg/ml (IL-6) or  mg/L (CRP) (95%CI) | P-Value |
| All participants | | | | | | |
| IL-6 | 4243 | -0.80 (-1.37, - 0.24) | 0.005 | 2960 | -0.26(-0.93, 0.41) | 0.446 |
| CRP | 4253 | -0.89 (-1.30, -0.49) | <0.001 | 2967 | -0.79(-1.31, -0.27) | 0.003 |
| Has Atopic Disorder | | | | | | |
| IL-6 | 1233 | -1.70 (-2.76, -0.64) | 0.002 | 983 | -1.04 (-2.24, 0.16) | 0.091 |
| CRP | 1238 | -0.93 (-1.63, -0.22) | 0.010 | 987 | -0.40 (-1.28, 0.47) | 0.367 |
| No Atopic Disorder | | | | | | |
| IL-6 | 2444 | -0.35 (-1.08, 0.39) | 0.355 | 1977 | 0.05 (-0.75, 0.86) | 0.897 |
| CRP | 2448 | -0.94 (-1.49, -0.39) | 0.001 | 1980 | -1.01 (-1.66, -0.36) | 0.002 |

^1^Adjusted for sex, BMI, maternal occupation, and household crowding

Online Supplementary Table S11: Association between quartiles of infection burden and serum IL-6 and CRP levels

| Inflammatory Marker/ Group | Infection Burden between 1.5 and 7.5 years (No. of Infections) | Sample Size, No. | In top third of inflammatory marker distributions  No. (%) | OR (95% CI) for being in the top third of inflammatory marker distribution at 9 years | | |
| --- | --- | --- | --- | --- | --- | --- |
|  |  |  |  | Unadjusted | | Adjusted^1^ |
| IL-6 | | | | | | |
|  | 1^st^ Quartile (0-3) | 1306 | 413 (31.62) | [reference] | [reference] | |
|  | 2^nd^ Quartile (4-5) | 1284 | 418 (32.55) | 1.04 (0.88, 1.23) | 1.12 (0.90, 1.39) | |
|  | 3^rd^ Quartile (6-7) | 998 | 332 (33.27) | 1.08 (0.90, 1.29) | 1.15 (0.92, 1.44) | |
|  | 4^th^ Quartile (8-19) | 1038 | 363 (34.97) | 1.16 (0.98, 1.38) | 1.06 (0.85, 1.33) | |
| CRP | | | | | | |
|  | 1^st^ Quartile (0-3) | 1306 | 402 (30.78) | [reference] | [reference] | |
|  | 2^nd^ Quartile (4-5) | 1284 | 409 (31.85) | 1.05 (0.89, 1.24) | 1.17 (0.92, 1.46) | |
|  | 3^rd^ Quartile (6-7) | 998 | 354 (35.47) | 1.24 (1.04,1.47) | 1.40 (1.10, 1.77) | |
|  | 4^th^ Quartile (8-19) | 1038 | 367 (35.36) | 1.23 (1.03, 1.46) | 1.09 (0.86, 1.39) | |
